# Symptomatic appendiceal intussusception—rare pitfall of the historical inversion technique

**DOI:** 10.1093/jscr/rjae086

**Published:** 2024-02-23

**Authors:** Xinyi Nan, Braden Pyle, Charlotte Kwik, Greg J Nolan

**Affiliations:** Department of General Surgery, Surgical & Critical Care Division, Gold Coast Hospital & Health Service, 1 Hospital Blvd, Southport, Queensland 4215, Australia; Colorectal Surgery, Department of General Surgery, Surgical & Critical Care Division, Gold Coast Hospital & Health Service, 1 Hospital Blvd, Southport, Queensland 4215, Australia; Colorectal Surgery, Department of General Surgery, Surgical & Critical Care Division, Gold Coast Hospital & Health Service, 1 Hospital Blvd, Southport, Queensland 4215, Australia; Colorectal Surgery, Department of General Surgery, Surgical & Critical Care Division, Gold Coast Hospital & Health Service, 1 Hospital Blvd, Southport, Queensland 4215, Australia

**Keywords:** appendiceal intussusception, appendiceal inversion, inversion-ligation technique, partial caecectomy

## Abstract

Appendiceal intussusception is a rare condition with an unknown incidence of clinical presentation, and an estimated incidence of 0.01% is based on a histological study only. It presents a diagnostic challenge with lack of standardized management strategies, and its description in literature is limited to case reports and series. Clinical presentation is often variable and nonspecific; it is uncommon to have a definitive preoperative diagnosis. Iatrogenic appendiceal intussusception can occur as a result of the historical simple inversion or inversion-ligation appendicectomy technique, but it is seldom reported to cause symptoms. We present a case of symptomatic appendiceal intussusception diagnosed preoperatively on both computed tomography and colonoscopy prior to proceeding with elective definitive surgery in a patient with no reported prior history of appendicectomy.

## Introduction

Appendiceal intussusception (AI) is a rare condition with an estimated incidence of 0.01% based on a histological study of appendicectomy specimens [1]; the true incidence of clinical presentation is unknown due to its scarcity. Its description in literature is limited to case reports and series, presenting a diagnostic challenge with lack of standardized management strategies [2]. Clinical presentation is often variable with nonspecific, acute, or chronic abdominal symptoms. Diagnosis has largely been made intraoperatively or histologically and it is uncommon to have a definitive preoperative diagnosis [2, 3]. Another differential diagnosis which can present essentially radiologically identical to AI is the simple inversion or inversion-ligation appendicectomy technique, which is performed historically during open surgeries for appendicitis or as a technique for incidental appendectomy to theoretically reduce the risk of peritoneal contamination [4].

We report a case of symptomatic AI diagnosed preoperatively on both computed tomography (CT) and colonoscopy prior to proceeding with an elective definitive surgery in a patient with no reported prior history of appendicectomy.

## Case presentation

**Figure 1:**
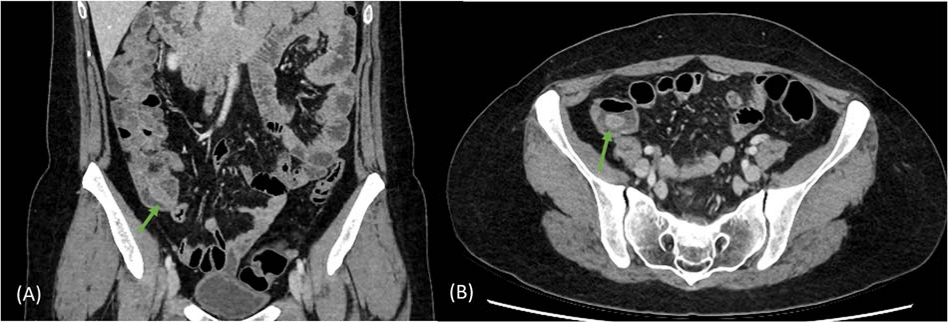
Coronal (A) and axial (B) views of CT scan demonstrating tubular rim enhancing structure in the caecal lumen with surrounding fluid, suspicious for invaginated appendix, which was otherwise not identified.

**Figure 2:**
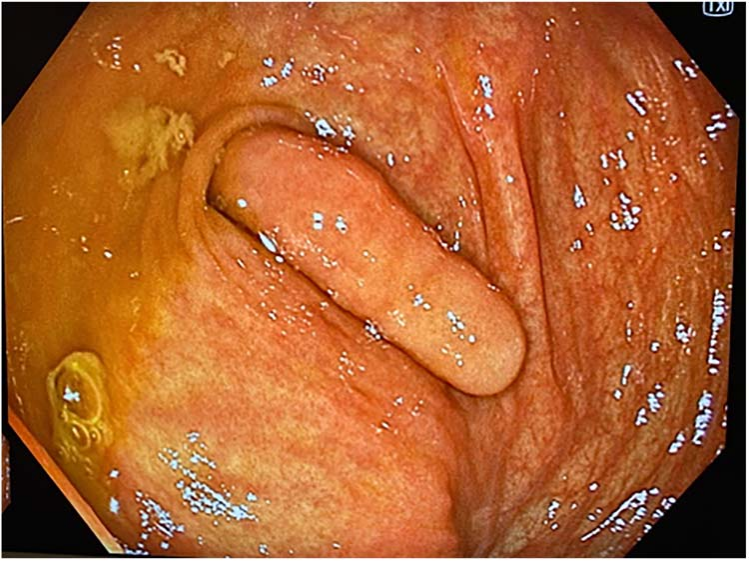
Colonoscopy image demonstrating a visible invaginated appendix within the caecal pole, with no other mucosal lesions or abnormalities seen.

**Figure 3:**
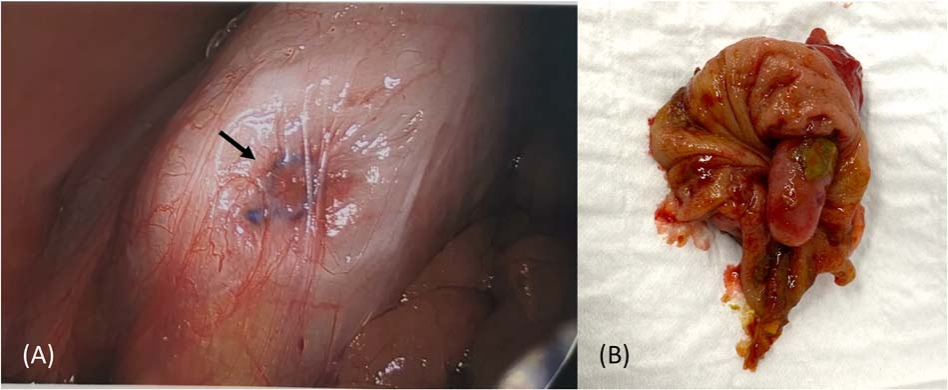
Intraoperative image of caecum with no visible appendix and suture material at the base (A), and partial caecetomy specimen showing an invaginated intact appendix (B).

A 41-year-old female presented to the emergency department with a 6-day history of nausea, diarrhoea, per rectal (PR) bleeding on straining, and generalized abdominal pain that was most prominent in the right iliac fossa (RIF). This is on the background of a previous similar episode 4 months ago which self-resolved after 3 days. Past medical history was significant for previous left oophorectomy via a right paramedian incision performed overseas and laparoscopic cholecystectomy. She had no reported history of appendicectomy. There was no history of allergies or other atopic skin conditions. On examination, the abdomen was soft and tender in the RIF without guarding or peritonism. Full blood count and biochemistry were unremarkable. Contrast enhanced CT of the abdomen and pelvis demonstrated a tubular rim enhancing structure in the caecal lumen with surrounding fluid, which was suspicious for invaginated appendix that was otherwise not identified (Fig. 1). She was conservatively managed with intravenous antibiotics and discharged home with oral antibiotics after 3 days. Outpatient colonoscopy performed 10 days postdischarge demonstrated a visible invaginated appendix within the caecal pole (Fig. 2). No other mucosal lesions or abnormalities were identified. On outpatient review, the patient reported ongoing right-sided abdominal pain without further PR bleeding and proceeded to a laparoscopic stapled caecectomy 4 days later. Intraoperatively, there were adhesions to the previous right paramedian incision. The appendix was within the caecum and appeared to have been tied there with suture material at the base (Fig. 3). Histopathology showed an appendix with eosinophilic granulomas and there was no evidence of helminth infection. The patient had an uneventful postoperative recovery process and was discharged home on Day 2 postoperation. She was well upon reviewed in the outpatient clinic 1 month later and was discharged.

## Discussion

Intussusception is defined as the invagination of a segment of intestine into an adjacent intestinal lumen caused by peristalsis, and it is more likely to occur if there is an intraluminal lesion acting as ‘lead point’ [5]. Intussusception in general is exceptionally rare in adults; however, AI has higher incidence in adults (76%) compared to children, and adult females were two times more affected [2]. Since its first description in 1858 by McKidd, just over 200 cases of AI have been reported [2, 6]. It is estimated that over 70% of cases are associated with either benign or malignant lesions, such as endometriosis, polyps, mucoceles, or foreign bodies [2, 4]. Iatrogenic intussusception of the appendix via the historical appendicectomy technique of simple inversion and ligation-inversion, described in the 1970–90s, can also present similarly on radiological imaging [4, 7].

Presenting symptoms of AI are variable and nonspecific, ranging from acute abdominal pain, nausea, and haematochezia to chronic intermittent symptoms [2]. AI can also remain asymptomatic and can be diagnosed incidentally intraoperatively or histologically. It is difficult to confidently diagnose the condition preoperatively; in fact, the first CT based diagnosis of AI was only reported in 2006 [8]. It can also be incidentally diagnosed on colonoscopy; however, vigilance is required to differentiate this uncommon finding to polyps as inadvertent biopsy or resection may lead to peritonitis [9].

No definitive guidelines to management of AI exists, given it is a rare entity. In the present case, a clear preoperative diagnosis based on a combination of CT imaging and colonoscopy was achieved, which helped to guide the prompt selection of an appropriate surgical resection. A variety of surgical options ranging from simple appendicectomy to right hemicolectomy exist, depending on the degree of suspicion for malignancy and the anatomy of the AI, which could be classified into five types according to the system proposed by McSwain [10]. The case report described here correlates with a McSwain Type 5, which is a complete invagination of the appendix into the caecum and a partial caecetomy was sufficient. Care should be taken to avoid operative complications, with such a case reported in literature where a small bowel obstruction secondary to ileocolic valve stenosis, resulting from a partial caecetomy for an incidental AI, required a further ileocecectomy [11].

Given the intraoperative finding of suture like material at the base of caecum (Fig. 3A), an intact inverted appendix, and histopathology not revealing another causative ‘lead point’, it appears the patient may have undergone an incidental appendectomy via the inversion-ligation technique during her previous left oophorectomy via a right paramedian incision. This technique involves inverting the skeletonized, intact appendix bluntly into the caecal lumen, ligating the remaining nubbin of tissue, and inverting this with a purse-string stitch [12]. It is not performed in the modern surgical era of laparoscopic appendicectomy and may not be familiar to younger clinicians. Although literature suggests that patients with a surgically inverted appendix are largely asymptomatic [9], the current case highlights it as a rare form of iatrogenic cause of symptomatic AI.

Symptomatic AI is a rare but important condition to consider for surgeons, endoscopists, and radiologists alike. This case demonstrates the safety of an elective interval surgery after full workup with CT and colonoscopy, and it highlights the importance of thorough past medical and surgical history taking to identify potential causes of AI.
